# Unveiling the devastations of *Streptococcus sanguinis* infective endocarditis masquerading as iron deficiency anaemia: a case report

**DOI:** 10.1093/ehjcr/ytae388

**Published:** 2024-08-01

**Authors:** Bing Wei Thaddeus Soh, Arifa Salim, Ruth O'Riordan, Patrick Owens, Sajjad Matiullah

**Affiliations:** Department of Cardiology, University Hospital Waterford, Dunmore Rd, Ballynakill, Waterford X91 ER8E, Ireland; Department of Infectious Diseases, University Hospital Waterford, Dunmore Rd, Ballynakill, Waterford X91 ER8E, Ireland; Department of Infectious Diseases, University Hospital Waterford, Dunmore Rd, Ballynakill, Waterford X91 ER8E, Ireland; Department of Cardiology, University Hospital Waterford, Dunmore Rd, Ballynakill, Waterford X91 ER8E, Ireland; Department of Cardiology, University Hospital Waterford, Dunmore Rd, Ballynakill, Waterford X91 ER8E, Ireland

**Keywords:** *Streptococcus sanguinis*, Infective endocarditis, Mycotic aneurysm, Aneurysm rupture, Multimodal imaging, Case report

## Abstract

**Background:**

Iron deficiency is the leading cause of anaemia worldwide and frequently observed in adolescent women, particularly those with eating disorders like anorexia nervosa. Consequently, clinicians may overlook iron deficiency anaemia, potentially missing a more serious diagnosis.

**Case summary:**

A 19-year-old woman was referred to the hospital by her general practitioner due to worsening symptomatic iron deficiency anaemia, despite treatment with oral iron supplementation. Her blood cultures consistently grew *Streptococcus sanguinis*, and an echocardiogram revealed vegetations on the mitral and tricuspid valves, confirming the diagnosis of infective endocarditis. Several systemic complications of varying acuity were identified, including a ruptured left common iliac artery aneurysm with active haemorrhage into the left psoas muscle, enlarging cerebral, hepatic, and right common femoral artery aneurysms, splenic infarction with abscess formation, and an infected left psoas muscle haematoma. Multimodal imaging and collaboration within the multidisciplinary endocarditis team were crucial for coordinating further evaluation and managing the complex array of peripheral lesions in infective endocarditis. The patient was discharged with a good clinical outcome after 81 days.

**Discussion:**

This case highlights the risks of overlooking iron deficiency anaemia in adolescent women with anorexia nervosa and the serious consequences of untreated complicated infective endocarditis. It emphasizes the need for thorough investigation of anorexia nervosa patients for infections due to their reduced clinical response, to ensure early diagnosis and treatment.

**Learning points**

- Immunocompromised anorexia nervosa patients exhibit a diminished clinical response to bacterial infections, often lacking fever or infective symptoms, thereby delaying the diagnosis and treatment of serious infections.
- In the context of anorexia nervosa, which commonly presents with iron deficiency anaemia in young women, comprehensive investigations for infections are warranted even when presenting symptoms are vague and align with anaemia.
- Multimodal imaging and collaboration within the ‘Endocarditis team’ are crucial for evaluating and managing the complex array of peripheral lesions in infective endocarditis.

## Introduction

Given the high global prevalence of anaemia (24.3%) and iron deficiency (ID) (15–25%) in adolescent women,^1,2^ clinicians may easily overlook the clinical significance of iron deficiency anaemia (IDA) in young women, particularly those with known eating disorders like anorexia nervosa (AN). This oversight can have catastrophic consequences, as AN patients are often immunocompromised, resulting in a reduced clinical response to bacterial infections,^3,4^ which in turn leads to delayed diagnosis and increased morbidity and mortality from these infections.^4^*Streptococcus sanguinis*, a member of the viridans group streptococci (VGS) reported as the second most frequent microorganism causing infective endocarditis (IE), is an abundant commensal bacteria in the mouth.^5^ Despite its perceived low pathogenicity, *S. sanguinis* can cause IE when it enters the bloodstream through routine activities like brushing.^5–7^ Here, we present an extreme case of *S. sanguinis* IE complicated by systemic mycotic aneurysms, necessitating multiple interventions in a young woman with IDA and AN.

## Summary figure

**Figure:**
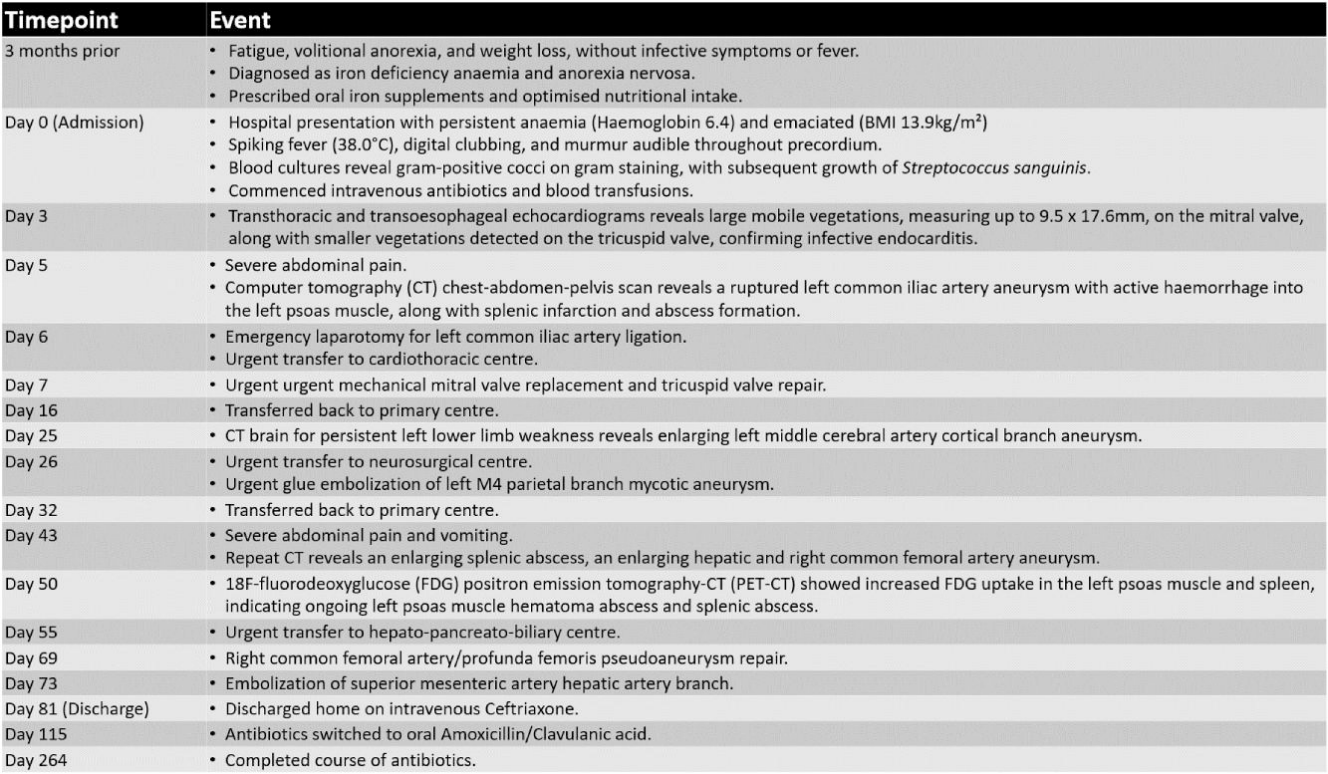


## Case presentation

A 19-year-old woman was referred to the emergency department by her general practitioner due to symptomatic refractory IDA. She had a four-month history of fatigue, volitional anorexia, and weight loss, but no infective symptoms or fever. Initial blood tests indicated IDA [haemoglobin 10.0 g/dL (reference range 12.0–15.0 g/dL), transferrin 2.0 g/L (reference range 2.0–3.6 g/L), transferrin saturation 10.0% (reference range 20.0–50.0%)], and she was treated accordingly. Despite three months of oral iron supplementation and nutritional rehabilitation, her symptoms persisted, and her anaemia worsened (haemoglobin 5.8 g/dL), prompting the referral. Apart from AN with retained menstruation, her medical history was unremarkable. Upon presentation, she exhibited a fever (38.0°C) and was tachycardic (145 beats per minute), with stable blood pressure (133/86 mmHg), oxygen saturation (98%), and respiratory rate (18 breaths per minute). She was clinically emaciated, with a BMI of 13.9 kg/m². Cardiovascular examination revealed bilateral digital clubbing and a systolic murmur audible throughout the precordium. Electrocardiogram revealed sinus tachycardia.

Initial laboratory investigation identified persistent IDA (haemoglobin 6.4 g/dL, transferrin 1.4 g/L, transferrin saturation 9.1%), elevated white blood cell (WBC) count (19.1 × 10^9^/L) (reference range 4.0–10.0 × 10^9^/L), C-reactive protein (104 mg/L) (reference range 0.0–5.0 mg/L), and lactate (4.1 mmol/L) (reference range 0.9–1.7 mmol/L) (*Table 1*). Three sets of blood cultures, drawn at 30 min intervals, consistently showed gram-positive cocci on gram staining. At this stage, the differential diagnosis included gram-positive sepsis with profound anaemia. She was promptly started on intravenous piperacillin/tazobactam, and an urgent blood transfusion was ordered.

**Table 1:** Laboratory investigation results from pre-hospital, emergency department presentation, and discharge

Investigation	Units	Normal range	Pre-hospital	Presentation	Discharge
Haemoglobin (Hb)	g/dL	12.0–15.0	10.0	6.4	13.4
Mean corpuscular volume (MCV)	fL	83.0–101.0	85.6	79.3	90.1
White blood cell (WBC)	×10^9^/L	4.0–10.0	13.8	19.1	6.5
Neutrophils	×10^9^/L	2.0–7.0	9.3	16.2	3.5
Platelets	×10^9^/L	150–400	410	470	332
C-reactive protein (CRP)	mg/L	0–5	7	104	<1
Urea	mmol/L	2.5–7.8	39	3.2	4.7
Creatinine	μmol/L	45–84	64	57	75
Iron	μmol/L	6.6–26	5.0	3.4	—
Transferrin	g/L	2.0–3.6	2.0	1.4	—
Transferrin saturation	%	20–50	10.0	9.1	—
Bilirubin	μmol/L	2–21	—	5.3	6.1
ALT	U/L	5–33	—	24	32
GGT	U/L	6–42	—	54	20
Albumin	g/L	35–50	—	28	45
pH	—	7.32–7.43	—	7.35	—
Lactate	mmol/L	0.9–1.7	—	4.1	—
Blood cultures	—	—	—	*S. sanguinis* × 3	—

Despite elevated white blood cell and neutrophil counts pre-hospital, the absence of infectious symptoms and fever, along with vague symptoms consistent with iron deficiency anaemia, led to the oversight of a more serious diagnosis, such as infection.

**Figure 1:**
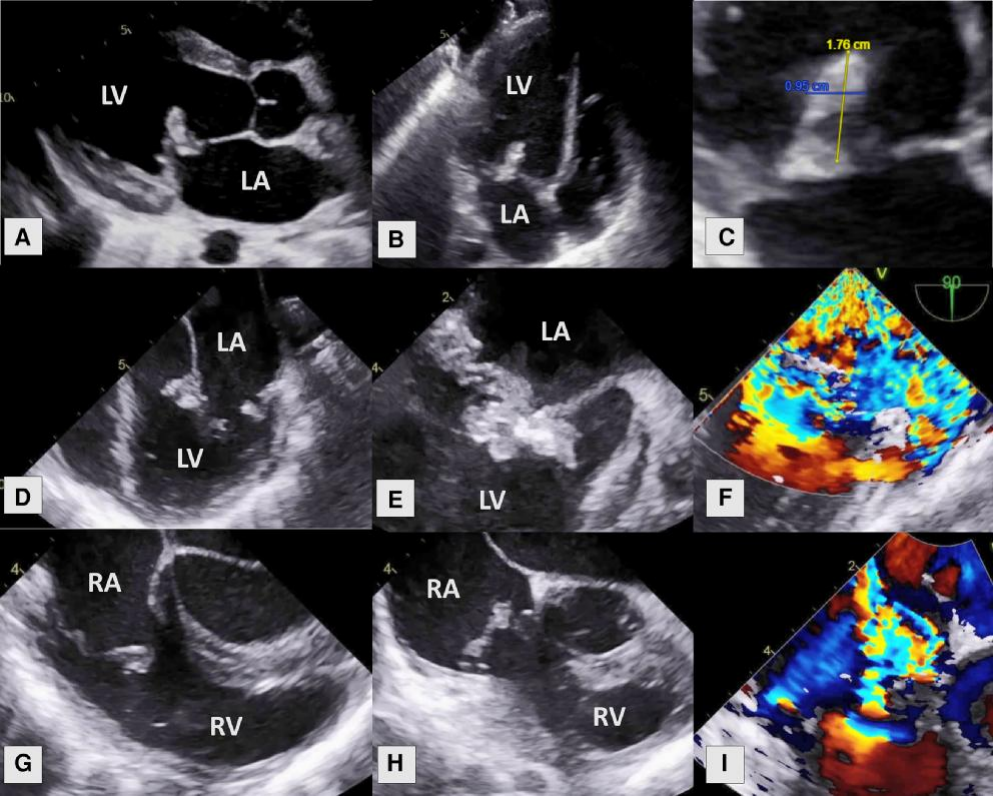
Transthoracic and transoesophageal echocardiogram view of MV and TV vegetations. (*A*) Parasternal long-axis view revealing large MV vegetation; (*B*) apical four-chamber view; (*C*) zoomed in on vegetation measuring 9.5 × 17.6 mm; (*D*) mid-oesophageal four-chamber view of large MV vegetation; (*E*) zoomed in on MV in end-diastole; (*F*) severe mitral regurgitation on colour Doppler; (*G*) mid-oesophageal right ventricle inflow–outflow view of TV vegetation in mid-diastole; (*H*) TV vegetation in mid-systole; (*I*) severe tricuspid regurgitation on colour Doppler. MV, mitral valve; TV, tricuspid valve; LA, left atrium; LV, left ventricle; RA, right atrium; RV, right ventricle.

Further investigations with transthoracic (TTE) and transoesophageal echocardiograms (TOE) revealed normal left ventricular size and function (LVEF > 60%), but also large mobile vegetations, measuring up to 9.5 × 17.6 mm on the mitral valve, along with smaller vegetations on the tricuspid valve, both associated severe regurgitation (*Figure 1*). Echocardiographic findings together with subsequent consistent growth of *S. sanguinis* on blood cultures confirmed the diagnosis of IE. Her antibiotics were escalated to intravenous ceftriaxone, vancomycin, and gentamycin.

**Figure 2:**
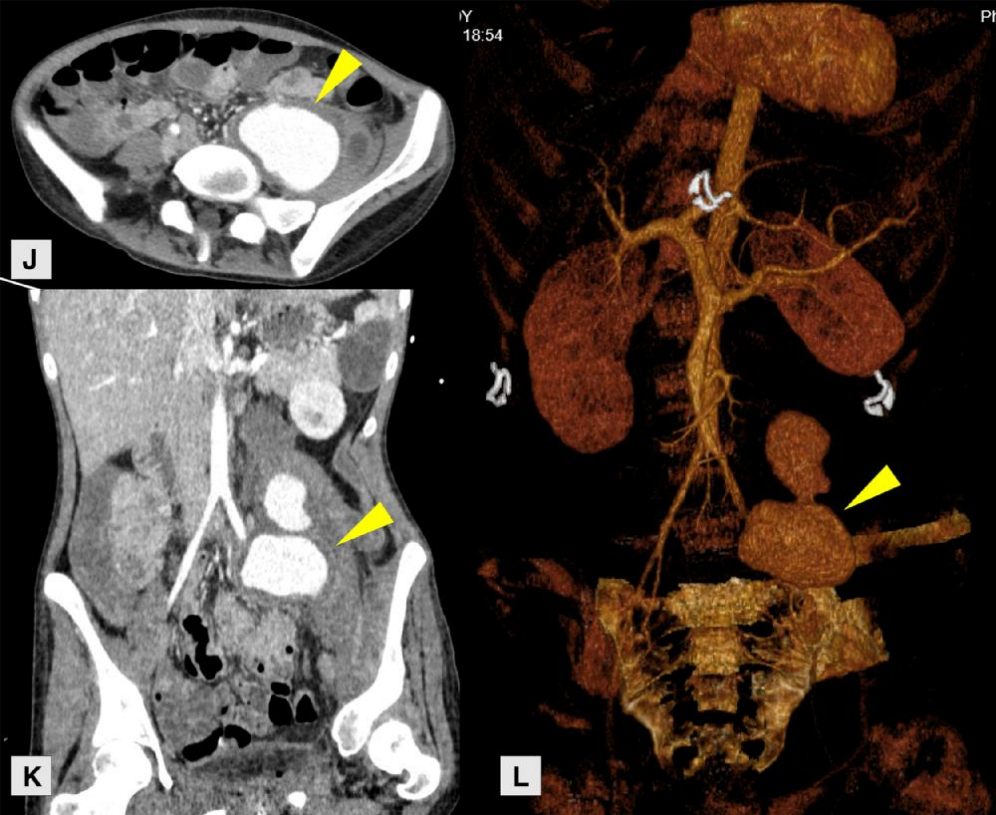
Computed tomographic angiography of the abdomen and pelvis revealing left internal iliac pseudoaneurysm rupture and haemorrhage into left psoas muscle (yellow arrowhead): (*J*) axial view; (*K*) coronal view; (*L*) 3D reconstruction of the left internal iliac pseudoaneurysm.

Although she received prompt intravenous antibiotics, her condition deteriorated, characterized by clinically significant abdominal pain and worsening anaemia, despite multiple blood transfusions. A computer tomography (CT)-chest-abdomen-pelvis scan was performed, revealing a ruptured left common iliac artery aneurysm with active haemorrhage into the left psoas muscle, along with splenic infarction and abscess (*Figure 2*). An emergency laparotomy was performed, and the left common iliac artery was ligated. She was subsequently transferred to a cardiothoracic centre for urgent mechanical mitral valve replacement and tricuspid valve repair.

**Figure 3:**
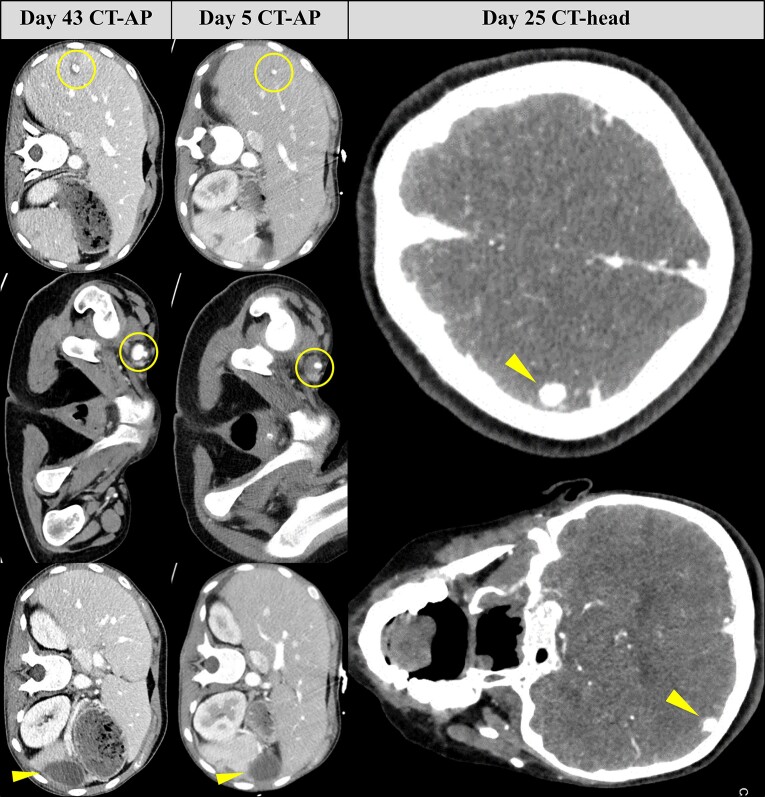
Brain and whole-body imaging with computed tomography (CT) demonstrating extracardiac complications. (Above) CT-head showing an enlarging left middle cerebral artery cortical branch aneurysm (yellow arrowhead). (Below) Side-by-side comparison of CT-abdomen-pelvis images illustrating the progression from Day 5 to Day 43: enlarging right hepatic lobe artery aneurysm (left yellow ring), enlarging right common femoral artery aneurysm (right yellow ring), and the evolution of splenic infarction to an enlarged splenic abscess (yellow arrowhead).

After successful surgeries without complications, she underwent intensive rehabilitation for physical deconditioning. Persistent left lower limb weakness prompted a CT-head, revealing an enlarging 9 mm left middle cerebral artery cortical branch aneurysm (*Figure 3*). The aneurysm had significantly increased in size despite antibiotics, thus necessitating an urgent neurosurgical centre transfer for glue embolization of the left M4 parietal branch mycotic aneurysm.

**Figure 4:**
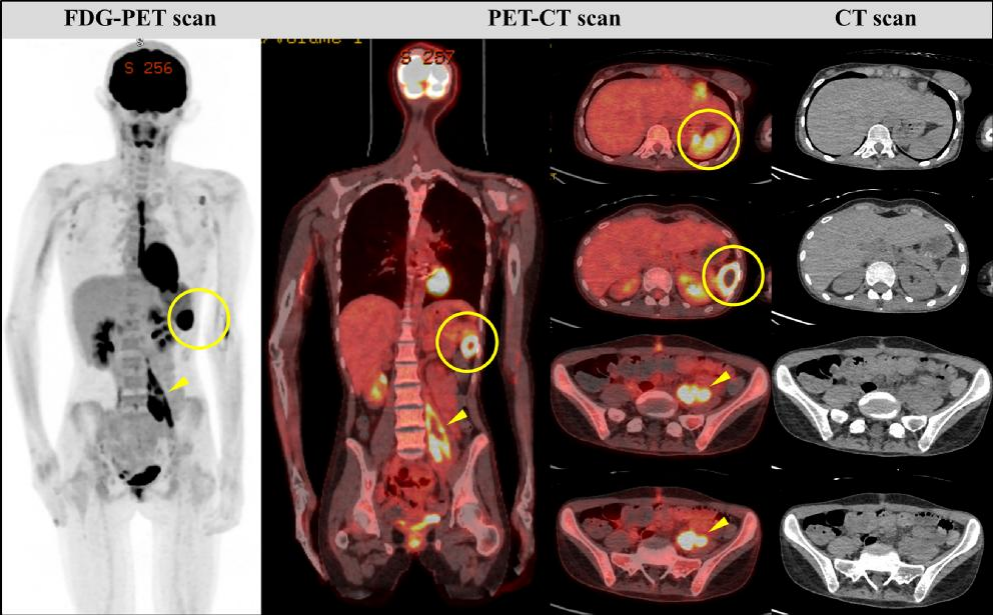
Whole-body PET-CT imaging reveals increased FDG uptake in the spleen (yellow ring) and left psoas muscle (yellow arrowhead). In the context of prior splenic infarction and abscess, and rupture of a left internal iliac pseudoaneurysm, these findings indicate ongoing splenic abscess and infected haematoma within the left psoas muscle. FDG, 18F-fluorodeoxyglucose; PET, positron emission tomography; CT, computed tomography.

Her troubles persisted with further abdominal pains and vomiting. A repeat CT revealed an enlarging splenic abscess, an enlarging 12 mm right hepatic lobe artery aneurysm, and an enlarging 19 mm right common femoral artery (CFA) aneurysm (*Figure 3*). Whole-body imaging with 18F-fluorodeoxyglucose (FDG) positron emission tomography-CT (PET-CT), indicated infected left psoas muscle haematoma and ongoing splenic abscess from increased FDG uptake (*Figure 4*).

After multidisciplinary ‘Endocarditis team’ discussion, the splenic abscess was managed conservatively rather than with a splenectomy, due to the reinfection risk posed by her prosthetic heart valve. Enlarging despite antibiotic treatment, the right hepatic lobe and CFA aneurysm posed a high risk of rupture. She was subsequently transferred to a hepato-pancreato-biliary centre where she underwent interventional radiology-guided embolization of the superior mesenteric artery hepatic artery branch and surgical repair of the right CFA aneurysm.

She was discharged from the hospital after 81 days, on warfarin and intravenous ceftriaxone arranged through outpatient parenteral antimicrobial therapy services. Follow-up was planned in multiple outpatient departments, including cardiology, infectious diseases, and the surgical specialties involved. Antibiotic prophylaxis for future oro-dental procedures was recommended according to the 2023 ESC guidelines.^8^

Showing steady recovery in the outpatient setting, her antibiotics were deescalated to oral amoxicillin/clavulanic acid on Day 115. Antibiotics were discontinued on Day 264 following surveillance CT confirmation of complete resolution of abscesses. She remained clinically well and free from clinical sequelae at 1-year follow-up.

## Discussion

Here, we present a devastating case of *S. sanguinis* IE, complicated by multiple systemic mycotic aneurysms, occurring in a young woman with IDA and AN, who did not exhibit infectious symptoms or fever until the infection had spread extensively.

The challenge of this case lies in the difficulty in diagnosing severe infections in AN patients early on due to the lack of typical infective symptoms and fever. The critical role of dietary protein in maintaining immune function is well established.^3^ Observational studies have shown that 80% of bacterially infected AN patients do not develop a fever response or symptoms, leading to delayed diagnosis and treatment, increasing the risk of infectious complications.^4^ Although the exact mechanism is unclear, acquired granulocyte dysfunction due to malnutrition may contribute to the absence of clinical symptoms despite elevated WBCs.^4,9^

The presence of IDA in a young woman adds to the complexity of this case. With the global prevalence of anaemia reported at 24.3% and ID being the most common cause,^1^ finding IDA in adolescent women can be expected.^2^ However, this may mislead clinicians from considering other critical issues, such as an underlying infection, particularly in the context of an immunocompromised patient with AN.

While a direct association between AN and IE has not been definitively established, several case reports have documented IE occurrences in young AN patients.^10–13^ These findings emphasize the importance of screening AN patients for infections, even when presenting with vague symptoms such as anaemia, to facilitate early diagnosis and treatment.

The diagnostic and management steps in our case demonstrate how the recommendations from the 2023 ESC guidelines are practically applied.^8^ TTE and TOE were used as the initial imaging modalities to confirm the diagnosis of IE (class I). Once symptomatic, brain and whole-body imaging (CT-head, CT-chest-abdomen-pelvis, and PET-CT) were performed to identify potential extracardiac lesions (class I). The patient was promptly managed with input from an ‘Endocarditis team’ and transferred to a cardiothoracic centre to improve outcomes (class I). Aneurysms that continued to increase in size despite antibiotics was appropriately treated with neurosurgical intervention (class I).

The successful outcome in our case highlights the effectiveness of a multidisciplinary ‘Endocarditis team’, comprising cardiology, infectious diseases, radiology, surgical specialities, and physiotherapy. This collaborative effort enabled the implementation of multiple advanced therapies, ultimately leading to the patient’s successful clinical outcome.

## Conclusion

Our case highlights the risks of overlooking IDA in patients with AN and demonstrates the severe consequences of untreated complicated IE. It highlights the importance of screening AN patients for infections, even with vague symptoms, to facilitate early diagnosis and treatment.

## Lead author biography


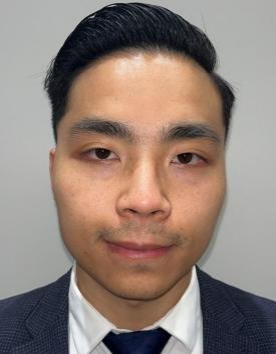


Dr. Bing Wei Thaddeus Soh, MB BCh BAO, graduated in 2019 from Trinity College Dublin, University of Dublin, Ireland. He has completed an MSc in Clinical Trials and is currently working as a cardiology specialist registrar while pursuing an MD thesis. Dr. Soh plans to further specialize with fellowship training in structural heart disease and interventional cardiology.

##  

**Consent**: The authors confirm that written informed consent for submission and publication of this case report including images has been obtained from the patient in line with the Committee on Publication Ethics (COPE) guidance.

**Funding:** There was no funding involved in this case report. This study did not receive any specific grant from any funding agency in the public, commercial, or not-for-profit sectors.

## Data Availability

There were no new data created or analysed in this study.
